# Fast Groupwise Registration Using Multi-Level and Multi-Resolution Graph Shrinkage

**DOI:** 10.1038/s41598-019-48491-9

**Published:** 2019-09-03

**Authors:** Pei Dong, Xiaohuan Cao, Pew-Thian Yap, Dinggang Shen

**Affiliations:** 1 Department of Radiology and BRIC, University of North Carolina at Chapel Hill, Chapel Hill, NC USA; 2 Department of Brain and Cognitive Engineering, Korea University, Seoul, Republic of Korea

**Keywords:** Biomedical engineering, Computational science, Computational neuroscience

## Abstract

Groupwise registration aligns a set of images to a common space. It can however be inefficient and ineffective when dealing with datasets with significant anatomical variations. To mitigate these problems, we propose a groupwise registration framework based on hierarchical multi-level and multi-resolution shrinkage of a graph set. First, to deal with datasets with complex inhomogeneous image distributions, we divide the images hierarchically into multiple clusters. Since the images in each cluster have similar appearances, they can be registered effectively. Second, we employ a multi-resolution strategy to reduce computational cost. Experimental results on two public datasets show that our proposed method yields state-of-the-art registration accuracy with significantly reduced computational time.

## Introduction

With advances in magnetic resonance imaging (MRI), a large number of brain images can be acquired for studying neurological disorders, such as Alzheimer’s disease (AD). Accurate spatial registration of these images is essential for detecting subtle disease-induced changes. Groupwise registration^1–8^ is often used to align the images to an unbiased common space.

The most intuitive means of aligning a population of images to a common space is by registering them to a pre-selected template. For example, images can be registered to the geometrical mean image^2^. In another study^4^, the template image was determined as the median of the image manifold, minimizing the sum of the geodesic distances from all images to the template. The registration paths of the images to the template were represented as a tree structure, and the images were registered to the template by composing the small deformations along these paths.

To avoid the bias associated with selection of a particular image as the template, Seghers *et al*.^9^ proposed a groupwise registration method to progressively warp each image to the group center by the average of the deformation fields of the image with respect to all other images. The congealing registration method^10–12^ can simultaneously drive all images to the group center by minimizing intensity-based stack entropies. Wang^13^ used image features to guide groupwise registration for better accuracy. But these methods are computational demanding, limiting their application to large datasets.

Registering images with large structural differences is challenging. Groupwise registration can be performed in an iterative manner by registering the images to a progressively updated group mean image^1,14^. For example, Joshi *et al*.^1^ proposed to compute a tentative group mean image by averaging all images after affine registration. The images were then warped to this mean image by using symmetric diffeomorphic Demons^15^. In each iteration, the tentative group mean image was updated by averaging all the warped images, and all the warped images were non-linearly registered to this updated group mean image. However, the accuracy of this method is limited by the blurriness of the tentative group mean image. To address this issue, Wu *et al*.^14^ proposed a method to improve the sharpness of the group mean image by using patch-based weighted averaging. A common limitation of these methods is that the registration accuracy of the images to the tentative group mean image might be affected by potentially large anatomical differences.

For accurate registration of datasets with large anatomical variations, the data distribution has to be taken into consideration. Wang *et al*.^6^ hierarchically clustered all images into several subgroups, forming a tree structure. Then, the subgroups are progressively and groupwisely registered using the tree structure. However, the way of how to accurately register the images within each subgroup remains untouched. Jia *et al*.^7^ proposed a method called ABSORB to gradually move each image towards the group center by using the average deformation field calculated from its neighboring images in the image manifold. Using a method called HUGS, Ying *et al*.^8^ represented image manifold using a graph, and then cast groupwise registration as a dynamic graph shrinkage problem. Although effective, this method is quite computationally expensive.

In this paper, we propose a hierarchical multi-level and multi-resolution graph-based framework for groupwise registration of heterogeneous datasets with high efficiency. To handle images with large anatomical variations, we propose to build a hierarchical multi-level vertex-labeled graph set to capture the complex inhomogeneous image distribution, where each vertex in the graph set is associated with an image and is labeled with a weight value. To boost the speed of registration, we employ a multi-resolution registration strategy. The main contributions of this work are summarized as follows:

1. We group images from an inhomogeneous distribution into multiple clusters so that the images can be registered hierarchically. The image distribution is represented using graphs: (1) the *intra-graph* is used to model the image distribution within each cluster, and (2) the *inter-graph* is used to model the image distribution across clusters. This hierarchical graph set allows us to model the image distribution locally and globally for accurate registration.
2. Based on the hierarchical graph set, each image is assigned a weight value, which is determined based on image similarity and indicates how fast that image can travel towards the group center. This weight can also help counter the imbalance of the numbers of images across clusters.
3. The hierarchical graph set allows registration to be performed very efficiently by composing small deformations of neighboring images. The speed is further improved by a multi-resolution strategy.

We evaluated the performance of our method using two public datasets: (1) LONI LPBA40 dataset consisting of brain images of normal young adults, and (2) Alzheimer’s Disease Neuroimaging Initiative (ADNI) dataset (*adni.loni.usc.edu*) with significant anatomical variations. Comparisons were performed with state-of-the-art groupwise registration methods, including GroupMean^1^, ABSORB, and HUGS.

In the rest of the paper, we describe our method (Section 2), report the experimental results (Section 3), provide further discussions (Section 4), and summarize the conclusions (Section 5).

## Method

**Figure 1:**
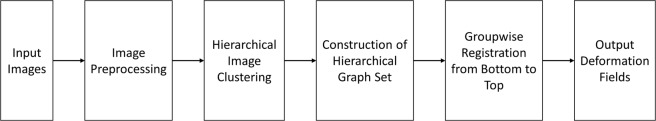
Overview of the registration framework.

An overview of the proposed method is shown in Fig. 1. We first hierarchically group the images into multiple clusters using affinity propagation (AP)^16^. Based on the clusters, a hierarchical graph set consisting of multiple inter-graphs and multiple intra-graphs is constructed to model respectively within-cluster and between-cluster image distributions. The registration process is then carried out by dynamic graph shrinkage using the relationships encoded in the inter- and intra-graphs. The final deformation field of each image with respect to the group center is then obtained by composing the deformation fields of neighboring images defined in the hierarchical graph set. A multi-resolution strategy is further employed for greater speed.

### Data preprocessing

Given a set of images ***I*** = {*I*_*i*_|*i* = 1, … *N*}, the preprocessing steps involve anterior commissure (AC) and posterior commissure (PC) correction, image resampling, image intensity inhomogeneity correction using N3 algorithm^17^, and skull striping using the Brain Extraction Tool (BET)^18^. Based on the sum of squared differences (SSD) computed in a tentative common space, the template is selected as follows:

1 $${I}_{t}=\mathop{{\rm{argmin}}}\limits_{I\in I}\mathop{\sum }\limits_{i=1}^{N}\,{\Vert {I}_{i}-I\Vert }^{2}$$

All images are linearly registered to the template using FLIRT^19^.

### Image clustering

We group the preprocessed images ${\boldsymbol{I}}{\boldsymbol{^{\prime} }}=\{{{I}_{i}}^{\text{'}}|i=1,\,\ldots \,N\}$ using affinity propagation (AP)^16^ into multiple clusters based on their proximity in the image manifold. Compared with conventional methods such as *k*-means^20^, the AP clustering method does not require a pre-specification of the number of clusters. AP clustering is based on an image similarity matrix *S*, where the diagonal elements are called preference values and the off-diagonal elements are called similarity values between image pairs. Each cluster is associated with an exemplar. An image ${{I}_{i}}^{\text{'}}$ is more likely to be chosen as the exemplar when its associated preference value *s*(*i*, *i*), *i* ∈ [1, *N*], is high. However, since we have no priori preference, we set the same preference value for all images. As suggested in the literature^16^, we set the preference value *s*(*i*, *i*) to the median of the similarity values. Each off-diagonal element *s*(*i*, *j*)(*i*, *j* ∈ [1, *N*], *i* ≠ *j*) measures the proximity of images ${{I}_{i}}^{\text{'}}$ and ${{I}_{j}}^{\text{'}}$ in the image manifold. Here, the *s*(*i*, *j*) is the negative sum of squared distance (SDD) between any two images, i.e., $s(i,j)=-\,{d}_{i,j}=-\,\Vert {{I}_{i}}^{\text{'}}-{{I}_{j}}^{\text{'}2}\Vert$.

**Figure 2:**
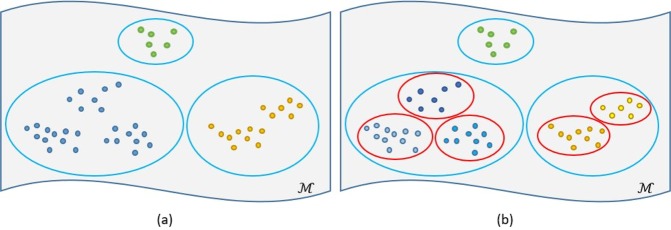
Hierarchical clustering of images in a manifold. The colored dots represent images in different clusters. (**a**) The first round of AP clustering. (**b**) Further clustering, where large clusters are further divided into smaller ones (red circles).

After the first round of AP clustering, the numbers of images in some clusters can be still large (see Fig. 2(a)). Thus, we iteratively apply AP clustering to further divide those clusters into smaller ones until the number of images in each cluster is below a pre-defined number (see Fig. 2(b)).

### Construction of a hierarchical graph set

**Figure 3:**
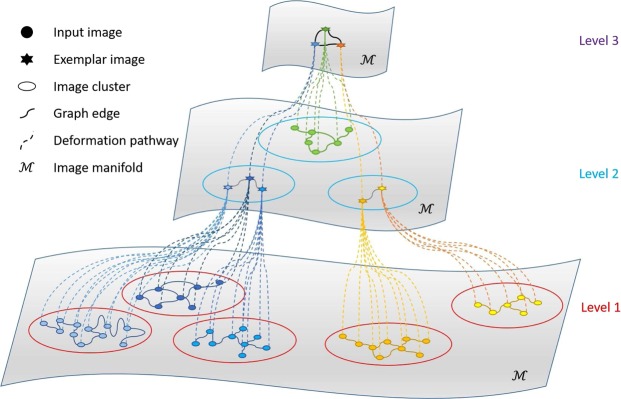
Hierarchical graph set. Colored dots denote images in different clusters. The stars denote the exemplar images. The circles mark the image clusters.

We construct a hierarchical vertex-labeled graph set, consisting of inter- and intra-graphs to model the image distribution within and between clusters (see Fig. 3). The graph set is constructed from the bottom-level clusters (e.g., Level 1 in Fig. 3) to top-level cluster (e.g., Level 3 in Fig. 3). An *intra-graph* is used for each cluster. An *inter-graph* is used for exemplars of different clusters.

The graph edges are determined based on the criteria used in a previous study^8^. First, all images should be linked in the graph so that they can be warped to their group center. Second, the number of graph edges need to be minimized for efficient graph shrinkage. Two criteria are used to assign the vertex labels for non-exemplar and exemplar images. Each vertex associated with a non-exemplar image (colored solid dots in Fig. 3) is labeled with the degree of the vertex (i.e., the number of edges connected to the vertex). Each vertex associated with an exemplar image (colored stars in Fig. 3) is labeled with the total vertex degree of its cluster. Each vertex is moved to the group center with different velocity according to its label, with large vertex label value moving slower than the one with smaller vertex label value.

### Groupwise registration by hierarchical graph shrinkage

With the hierarchical graph set, groupwise registration can be solved by using dynamically shrinking a series of graphs, starting from the graphs at the bottom level to the graph on the top level. For each graph shrinkage, we employ the HUGS method^8^ to warp all images to their group center. The HUGS method, which uses the simple graph shrinkage to solve the groupwise registration problem, is briefly described below.

#### Simple graph shrinkage

Given a set of the preprocessed image set ***I***′ within an image cluster, all the images ${\{{{I}_{i}}^{\text{'}}({t}_{0})\}}_{i=1}^{N}$ are sitting on the high-dimensional image manifold at the time *t*_0_. The topology of their distribution is described by the constructed labeled graph *G* = {*V*, *E*, *L*}, where the graph edge *e* ∈ *E* encodes the connectivity between vertices in graph vertex set *V* and each vertex label *l* ∈ *L* equals to the vertex degree. Particularly, a graph edge *e*_*ij*_ = 1 means images ${{I}_{i}}^{\text{'}}$ and ${{I}_{j}}^{\text{'}}$ are connected, while *e*_*ij*_ = 0 means no connection between images ${{I}_{i}}^{\text{'}}$ and ${{I}_{j}}^{\text{'}}$. A velocity vector *v*_*ij*_(*t*) is associated with each edge, where *v*_*ij*_(*t*) > 0 if *e*_*ij*_ = 1 and *v*_*ij*_(*t*) = 0 otherwise. Here, the velocity vector *v*_*ij*_(*t*) is associated to the graph edge *e*_*ij*_ to calculate the geodesic distance from the image ${I}_{i}^{\text{'}}(t)$ to the image ${I}_{j}^{\text{'}}(t)$ by integrating along the velocity vector. Graph shrinkage aims to minimize the objective function *F*(*t*) defined below as the summation of the geodesic distances between the linked images in the graph *G*:

2 $$F(t)=\mathop{\sum }\limits_{i,j=1}^{N}\,{e}_{ij}{d}^{2}({I}_{i}^{\text{'}}(t),{I}_{j}^{\text{'}}(t))=\mathop{\sum }\limits_{i,j=1}^{N}\,{e}_{ij}{\Vert {v}_{ij}(t)\Vert }^{2}$$

where *d*(*I*_*i*_(*t*), *I*_*j*_(*t*)) is the geodesic distance between images ${{I}_{i}}^{\text{'}}$ and ${{I}_{j}}^{\text{'}}$ on the image manifold.

The graph shrinkage process is implemented dynamically, such that each image at time point *t*_*n*_(*n* = 0, 1, 2, …, *K*, with *t*_0_ = 0, *t*_1_ = *t*_0_ + Δ*t*_1_, …, *t*_*K*_ = *t*_*K*−1_ + Δ*t*_*K*_) in the graph is deformed from ${I}_{i}^{\text{'}}({t}_{n})$ to ${I}_{i}^{\text{'}}({t}_{n}+{\rm{\Delta }}{t}_{n})$ with the decrease of overall geodesic distance described by *F*(*t*), while maintaining the topology of the graph. Since *F*(*t*) is a strictly and monotonically decreasing function with the increment of time^8^, all images can be warped to their group center with *K* iterations. Specifically, at time point *t*_*n*_, each image ${I}_{i}^{\text{'}}({t}_{n})$ is moving along the direction of the averaged velocity vector ${\hat{v}}_{i}({t}_{n})$ calculated with its connected neighbors in the graph *G*,

3 $${\hat{v}}_{i}({t}_{n})=\frac{1}{{l}_{i}}\mathop{\sum }\limits_{j=1}^{N}\,{e}_{ij}{v}_{ij}({t}_{n})$$

where *v*_*ij*_(*t*_*n*_) is the velocity vector between the image ${I}_{i}^{\text{'}}$ and its connected image ${I}_{j}^{\text{'}}$, and *l*_*i*_ is the image label of ${I}_{j}^{\text{'}}$ that denotes the vertex degree of ${I}_{i}^{\text{'}}$ in the graph. By using the diffeomorphism Demons^15^, the deformation field between image ${I}_{i}^{\text{'}}({t}_{n})$ and deformed image ${I}_{i}^{\text{'}}({t}_{n}+{\rm{\Delta }}{t}_{n})$ can be calculated as $\exp ({\rm{\Delta }}{t}_{n}\,\cdot \,{\hat{v}}_{i}({t}_{n}))$ ^21^, where the time increment Δ*t*_*n*_ is calculated by

4 $${\rm{\Delta }}{t}_{n}=\,min\{\frac{1}{ma{x}_{i}\Vert {\hat{v}}_{i}({t}_{n})\Vert },\,\frac{{\sum }_{i=1}^{N}\,{l}_{i}{\Vert {\hat{v}}_{i}({t}_{n})\Vert }^{2}}{{\sum }_{i=1}^{N}\,({l}_{i}+1){\Vert {\hat{v}}_{i}({t}_{n})\Vert }^{2}}\}.$$

Finally, the overall deformation field *φ*_*i*_, which warps the image ${I}_{i}^{\text{'}}({t}_{0})$ to the group center of this image cluster, can be calculated by concatenating each part of the deformation field segments at each time point:

5 $${\phi }_{i}=exp({\rm{\Delta }}{t}_{k}\cdot {\hat{v}}_{i}({t}_{k}))\circ \cdots \circ \,exp({\rm{\Delta }}{t}_{1}\cdot {\hat{v}}_{i}({t}_{1}))\circ \,exp({\rm{\Delta }}{t}_{0}\cdot {\hat{v}}_{i}({t}_{0}))$$

where the symbol ° denotes the composition of the deformation fields.

#### Hierarchical graph set shrinkage

**Figure 4:**
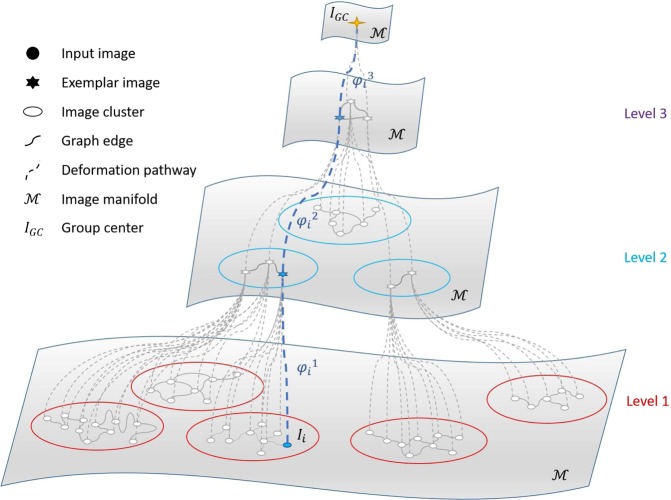
Hierarchical graph shrinkage. The whole deformation pathway ***ψ***_***i***_ of image ***I***_***i***_ can be obtained by the composition of deformation fields (${{{\boldsymbol{\phi }}}_{{\boldsymbol{i}}}}^{{\bf{1}}}\,\circ \,{{{\boldsymbol{\phi }}}_{{\boldsymbol{i}}}}^{{\bf{2}}}\,\circ \,{{{\boldsymbol{\phi }}}_{{\boldsymbol{i}}}}^{{\bf{3}}}$) from the bottom level to the top level.

Groupwise registration for the image set is performed sequentially through each image cluster via the hierarchical graph set from the bottom level towards the top level. Specifically, as illustrated in Fig. 4, for a certain image ${I}_{i}^{\text{'}}$ located at a bottom group at the first level (bottom level), we first obtain the deformation field ${{\phi }_{i}}^{1}$ using the simple graph shrinkage, which warps the image ${I}_{i}^{\text{'}}$ to the group center at the first level. Then, a warped image with the highest connection number in the local graph is selected as the exemplar image to participate the graph shrinkage in the group of the upper level. It should be noted that the exemplar is selected from one of the warped images, which does not involve calculating the mean image of the warped image set, which could undermine the registration accuracy at a higher level. By using the graph shrinkage method on the graph at the upper level, we can obtain the deformation field that warps the exemplar image to the group center at the next upper level. In this way, by performing the graph shrinkage method sequentially at different groups from the bottom level to the top level in the hierarchical structure, a set of deformation fields ${{\boldsymbol{\phi }}}_{i}=\{{{\phi }_{i}}^{p}|p=1,\,\ldots ,\,P(i)\}$ can be obtained. Finally, the deformation pathway *ψ*_*i*_, which warp the image ${I}_{i}^{\text{'}}$ to the group center of the whole image set *I*_*GC*_ can be obtained by composing each part of the deformation field in ***φ***_*i*_

6 $${\psi }_{i}={{\phi }_{i}}^{1}\,\circ \,{{\phi }_{i}}^{2}\,\circ \,\cdots \,\circ \,{{\phi }_{i}}^{P(i)}$$

In this way, the deformation field set **Ψ** = {*ψ*_*i*_|*i* = 1, …, *N*} can be obtained, where every image can be warped to the final group center *I*_*GC*_.

### Fast registration by using multi-resolution strategy

To further boost the registration speed, we employ the multi-resolution strategy to register the whole image set in a coarse-to-fine manner. Instead of registering all images in their original size, they are down-sampled multiple times. Registration is then performed successively from the coarse level (low resolution) to the fine level (original resolution).

## Experiments and Results

In the following, we will demonstrate the performance of our method on real brain images based on two public datasets in comparison with the state-of-the-art groupwise registration methods. We used two ways to evaluate the groupwise registration performance: (1) the volumetric overlap for the same ROIs or tissues after warping to the groupmean image, e.g., Dice Ratio, and (2) the sharpness of the group mean image. Two datasets were used to validate our proposed method. First, the experiment employed the LONI LPBA40 dataset^22^. This dataset contains 40 young adult brain MR images with 54 manually labeled region of interest (ROIs). Since all 40 brain images are all collected from the healthy and young adult subjects (with an average age of 29 years), the image anatomical/appearance variations were relatively small. The second experiment used the ADNI dataset, and here we randomly selected 100 subjects; among them, 50 subjects were selected from the normal control group, and 50 subjects were selected with Alzheimer’s disease (AD). This dataset had white matter (WM), gray matter (GM) and cerebrospinal fluid (CSF) tissues segmentation maps, which can be used to evaluate registration performance. This dataset has large anatomical/appearance variations due to the disease.

Three groupwise registration methods were used as the comparison methods, i.e., (1) the conventional groupmean registration method^1^, (2) ABSORB method^7^, and (3) HUGS method^8^. It is worth noting that, in order to compare fairly for all the methods, we use the symmetric diffeomorphic Demons^15^ to perform the pairwise registration involved in all groupwise registration methods. In addition, we conducted all experiments based on the same computing environment with Intel Xeon E7-4850, 2.0 GHz, ten cores CPU, and 256 GB/1066 MHz RAM.

To quantitatively evaluate our method, we employed the Dice ratio to measure the overlap degree of each brain ROI or tissue segmentation map after performing groupwise registration. The Dice ratio is defined as:

7 $$Dice=2\times \frac{|A\cap B|}{|A|+|B|}$$

where *A* and *B* denote the two corresponding brain ROIs in the two aligned brain images, respectively. Particularly, for groupwise registration, since the final group mean was not an image from the dataset, the label map of the group mean image was generated by majority voting among all the warped label images. The Dice ratio of each ROI in each certain registered image is then calculated *with respect to* the label image in the group mean space.

### Experiments on LONI LPBA40 dataset

**Figure 5:**
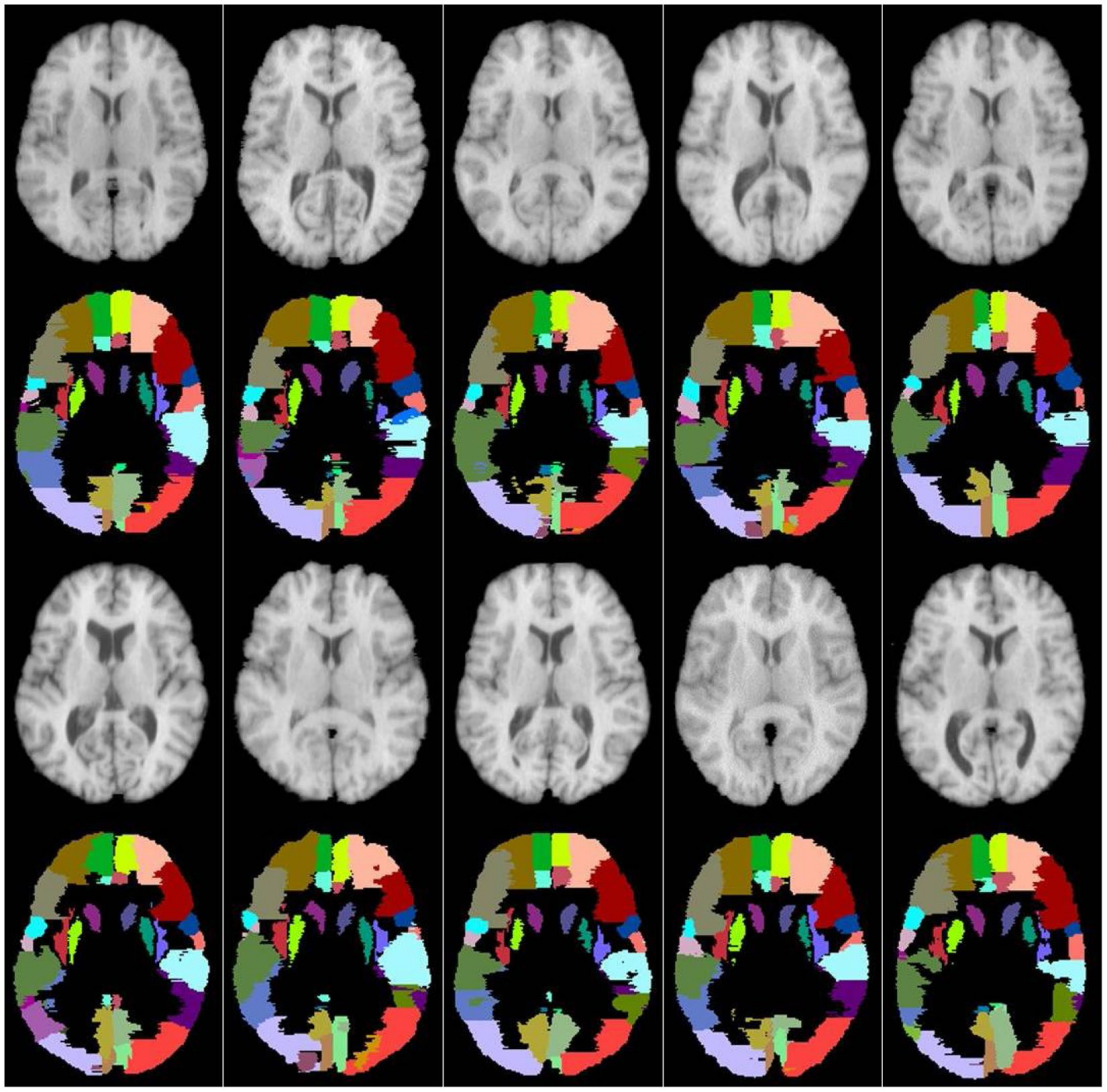
Typical examples of linearly aligned MR brain images and their corresponding labeled regions from LONI LPBA40 dataset.

In the first experiment, we evaluated the performance of our groupwise registration on LONI LPBA40 dataset^22^. After preprocessing (details provided in Section 2.1), all the images were linearly aligned. Typical MR images and their corresponding labels are shown in Fig. 5.

**Figure 6:**
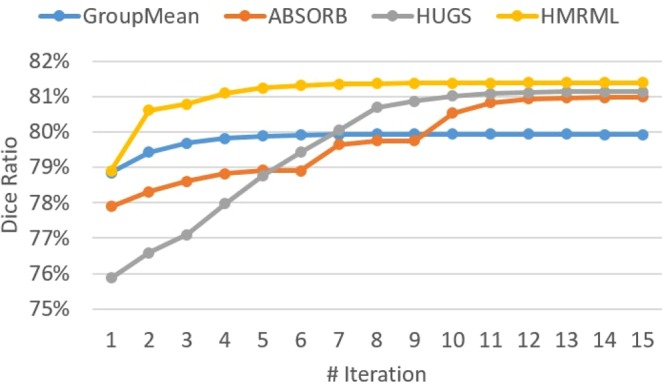
The evolution curves of the overall weighted Dice ratio for registering 40 subjects (each with 54 brain ROIs) on LONI LPBA40 dataset, using four groupwise registration methods.

To quantitatively compare both the efficiency and the accuracy of our method (HMRML) with other state-of-the-art groupwise registration methods, we first compared the maximum performance of each method, and then we compared the computational time of each method for reaching the maximum performance. In Fig. 6, we report the evolution curves of the overall weighted Dice ratios on 54 brain ROIs. The maximum performance of each method was determined based on the Dice ratio on the evolution curve at the iteration where the increase of slope ratio is smaller than 0.001. From Fig. 6, we can observe that the conventional GroupMean method reaches its maximum performance of 79.8% at the 4^th^ iteration, the ABSORB method reaches its maximum performance of 80.9% at the 12^th^ iteration, the HUGS method reaches its maximum performance of 81.0% at the 10^th^ iteration, and our method reaches its maximum performance of 81.2% at the 5^th^ iteration.

**Figure 7:**
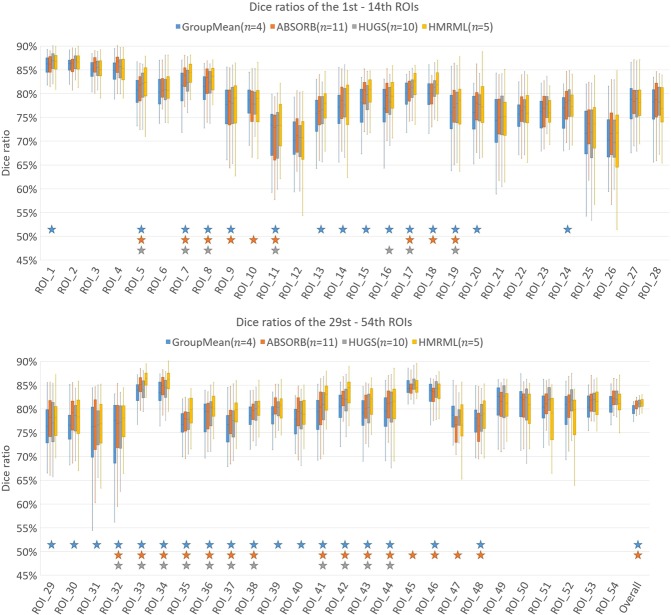
Box plots of Dice ratios of 54 brain ROIs of LONI LPBA40 dataset using our method (HMRML) and other three groupwise registration methods. ***n*** denotes the number of iterations for reaching the maximum registration accuracy. The blue, orange and grey stars mark statistically significantly improvements of our methods over GroupMean, ABSORB and HUGS, respectively: The names of the 54 ROIs are as follows: 1–2: L/R superior frontal gyrus, 3–4: L/R middle frontal gyrus, 5–6: L/R inferior frontal gyrus, 7–8: L/R precentral gyrus, 9–10: L/R middle orbitofrontal gyrus, 11/12: L/R lateral orbitofrontal gyrus, 13–14: L/R gyrus rectus, 15–16: L/R postcentral gyrus, 17–18: L/R superior parietal gyrus, 19–20: L/R supramarginal gyrus, 21–22: L/R angular gyrus, 23–24: L/R precuneus, 25–26: L/R superior occipital gyrus, 27–28: L/R middle occipital gyrus, 29–30: L/R inferior occipital gyrus, 31–32: L/R cuneus, 33–34: L/R superior temporal gyrus, 35–36: L/R middle temporal gyrus, 37–38: L/R inferior temporal gyrus, 39–40: L/R parahippocampal gyrus, 41–42: L/R lingual gyrus, 43–44: L/R fusiform gyrus, 45–46: L/R insular cortex, 47–48: L/R cingulate gyrus, 49–50: L/R caudate, 51–52: L/R putamen, 53–54: L/R hippocampus.

**Figure 8:**
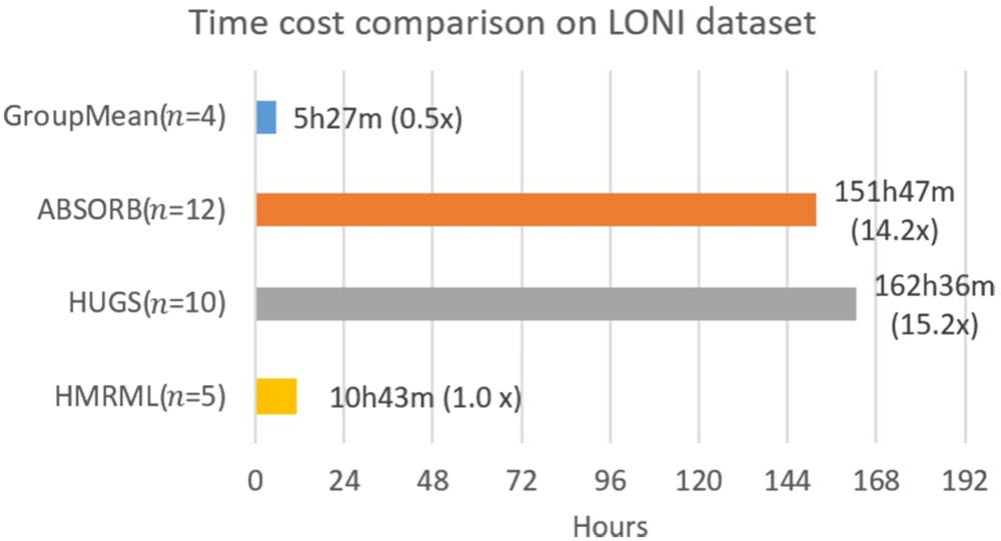
Computational times for registering 40 images on LONI LPBA40 dataset, computed based on the time required to achieving the maximum registration performance. ***n*** denotes the number of iterations needed for reaching the maximum registration accuracy.

In Fig. 7, we report the detailed registration results of our method and the other three comparison methods for all 54 brain ROIs. The colored stars mark statistically significant improvements (*t*-test, *p* < 0.05). It can be observed that our method yields significant improvement over the comparison methods for most ROIs. Also, our method achieves best overall performance with significant improvements compared with GroupMean and ABSORB, and is also comparable to HUGS. Figure 8 provides the computational time of each method for reaching their best registration performance. It can be observed that our method reduces the computational time significantly. Compared with HUGS method, to reach competitive performance, our method is about 15 times faster.

### Experiments on ADNI dataset

**Figure 9:**
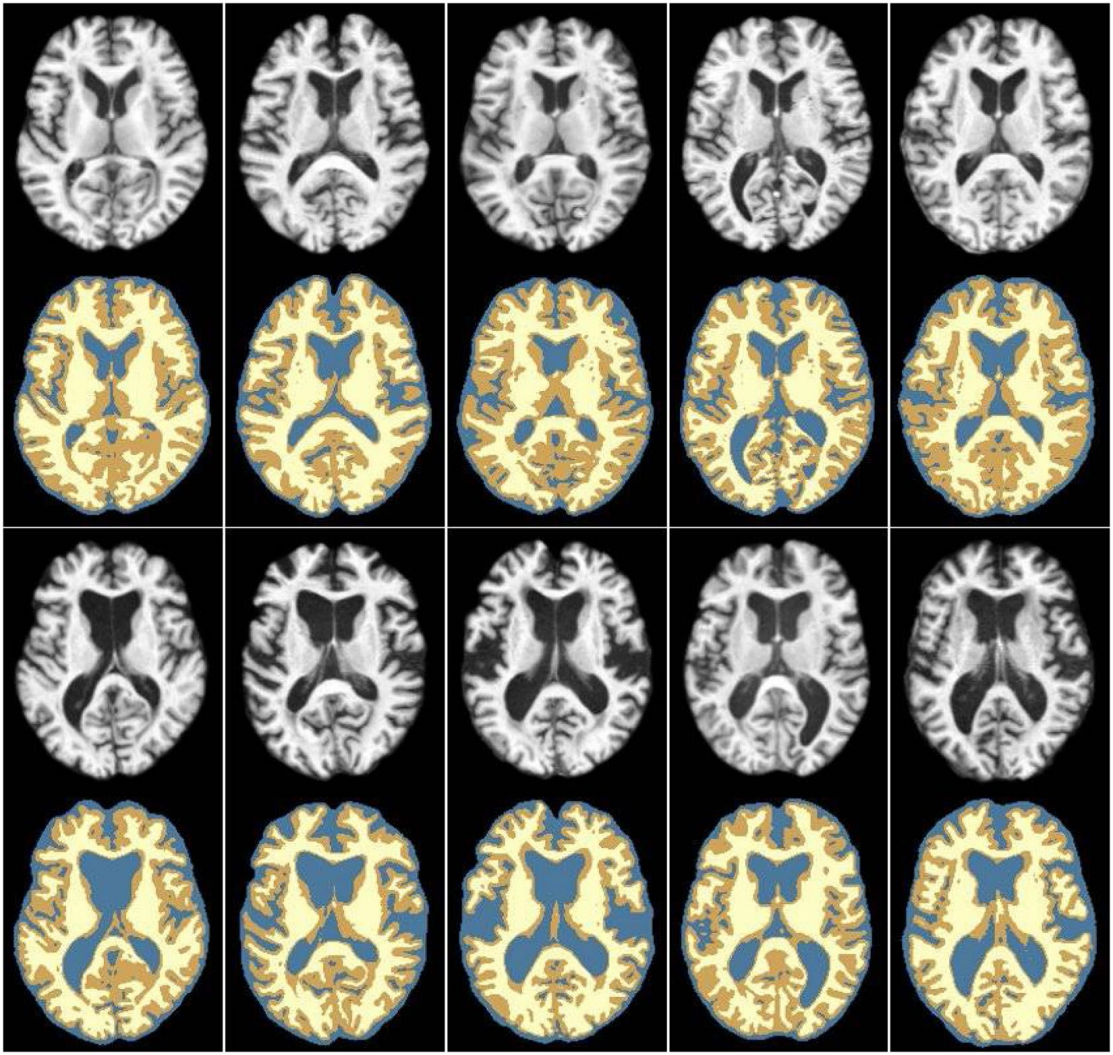
Typical examples of MR brain images and their corresponding tissue labels from ADNI dataset. The GM is labeled with darker yellow; the WM is labeled with lighter yellow, and the CSF is labeled with blue. The subjects in the first two rows are selected from the NC group, and the subjects in the last two rows are selected from the AD group.

In the second experiment, we evaluated the performance of our proposed method on a set of elderly brain images from the ADNI dataset, where the brain anatomical variations are large. After image preprocessing, all images were linearly aligned to the selected template image *I*_*t*_ calculated from Eq. (1), using the FSL FLIRT^19^. Then, all the images were cropped to the size of 196 × 164 × 176 to reduce image background. Next, each image was segmented into gray matter (GM), white matter (WM) and cerebrospinal fluid (CSF) using FAST^23^. These tissue segmentations were manually corrected with visual inspection and were regarded as the ground truth for evaluating the registration performance. In Fig. 9, the top two rows and bottom two rows show typical images and their corresponding label images randomly selected from both normal control (NC) group and AD group, respectively.

**Figure 10:**
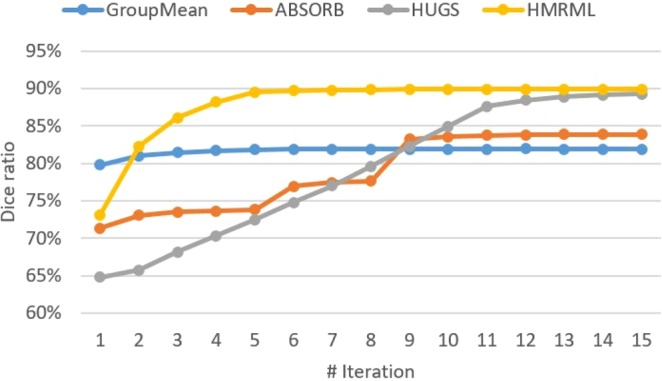
The evolution of the overall Dice ratio for the 54 brain ROIs during groupwise registration on the ADNI dataset.

**Figure 11:**
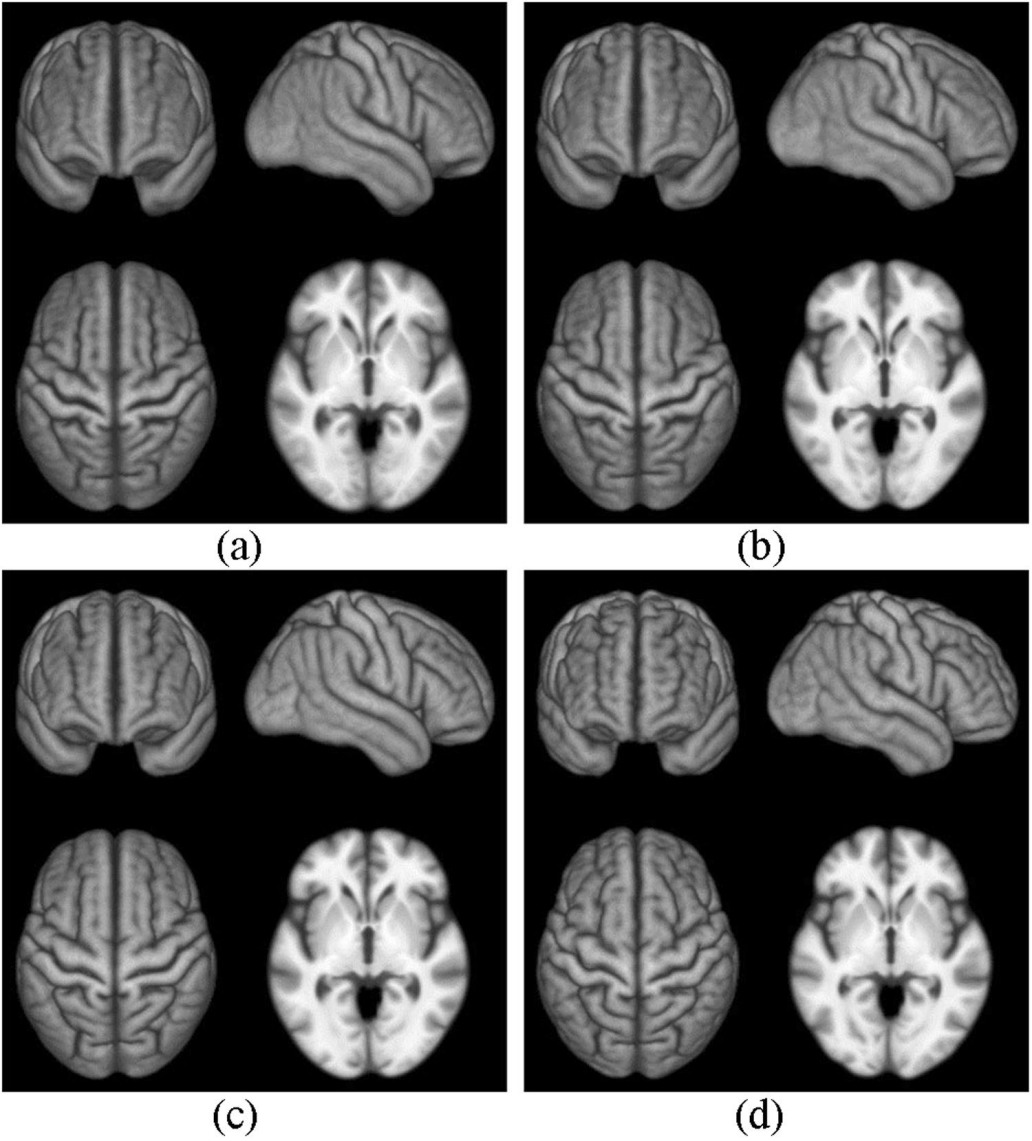
Group mean images given by (**a**) GroupMean, **(b**) ABSORB, (**c**) HUGS, and (**d**) our method.

**Figure 12:**
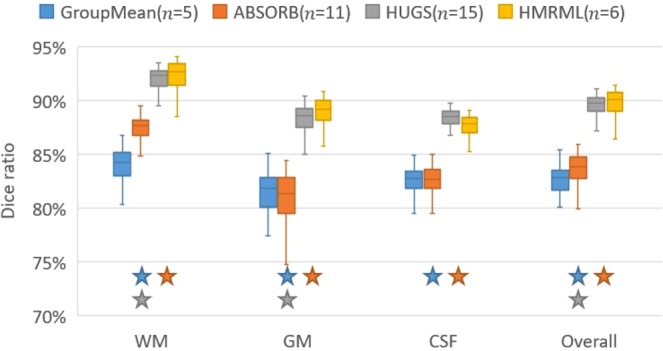
Box plots of Dice ratios of different brain tissue types given by the four methods on 100 warped images from the ADNI dataset. The blue, orange, gray and yellow stars indicate that our method yields a significant improvement in terms of Dice ratio over GroupMean, ABSORB, and HUGS on certain brain tissue type.

**Figure 13:**
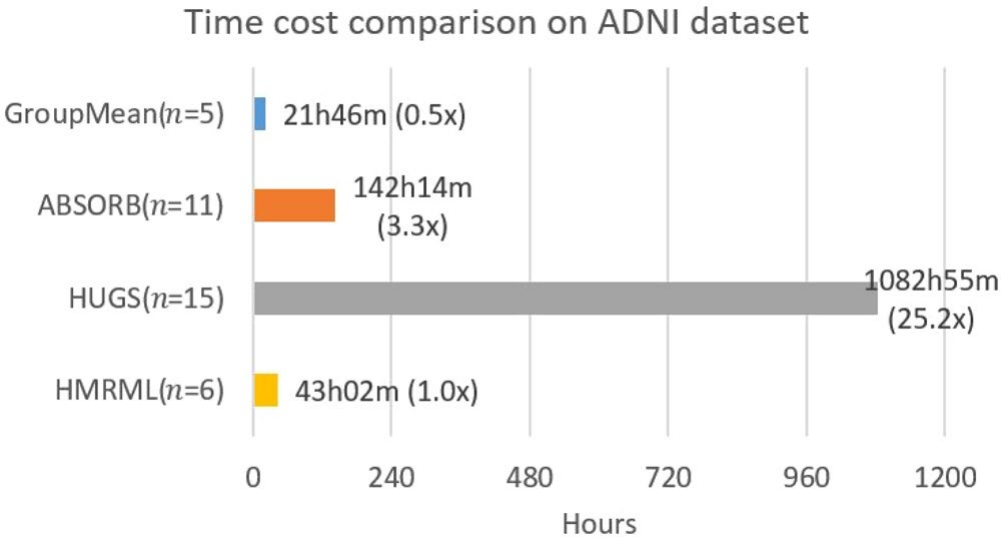
Time cost comparison between our method and other methods for registering 100 images from on ADNI dataset based on the time required to achieve maximum registration performance.

To quantitatively illustrate the performance of our method, we showed the evolution curves of the overall weighted Dice ratio of the brain tissues after groupwise registration in Fig. 10. It can be observed that GroupMean, ABSORB, HUGS and our method (HMRML) attain their maximum performance at the 5^th^ iteration, 11^th^ iteration, 15^th^ iteration, and 6^th^ iteration, respectively. Figure 11 show the group mean images of the four groupwise registration methods in achieving their maximum registration performance. It can be observed that the group mean image of our method showed much more detailed anatomical structures compared to other methods. In Fig. 12, we compare the maximum groupwise registration performance of our method with the other three methods. Our method achieves the best overall performance compared with other methods, with statistically significant improvements. Specifically, the overall Dice ratio for the brain tissues is 81.8% by the conventional GroupMean method, 83.7% by ABSORB method, 89.3% by HUGS method and 89.7% by our proposed method. Particularly, our method significantly outperforms all other methods for GM and WM. Besides improvements in terms of accuracy, Fig. 13 also shows that our method reduces the computational time significantly, especially when compared with HUGS, by a factor of more than 25.

## Discussion

**Figure 14:**
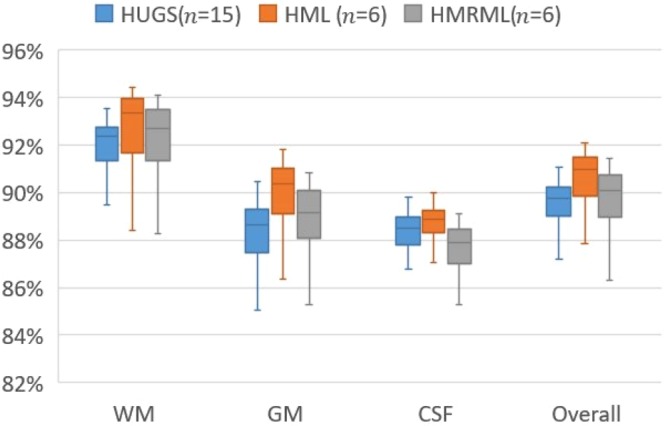
Box plots of Dice ratios of different brain tissue types given by the three comparison methods on 100 warped images from the ADNI dataset.

**Figure 15:**
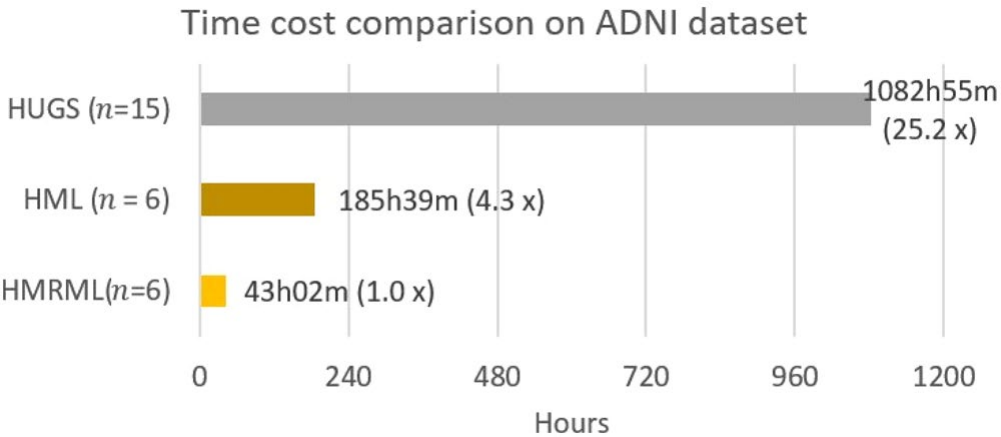
Time cost comparison between the method without hierarchical graph set and multi-resolution strategy (HUGS), the method with only hierarchical graph set shrinkage (HML), and the method with both hierarchical graph set shrinkage and multi-resolution strategy (HMRML).

Our method yields greater improvements in both registration accuracy and efficiency for the ADNI dataset compared with LONI LPBA40, due to the greater anatomical variation. The effectiveness of our method can be attributed to two factors: (i) A hierarchical graph set for registration using dynamic graph shrinkage, and (ii) Multi-resolution registration for speed. We evaluate the effectiveness of the proposed strategies using 100 images from the ADNI dataset in three different settings: (1) Single graph shrinkage (HUGS method); (2) Hierarchical multi-level graph set without deploying multi-resolution strategy (HML); and (3) Full strategy (HMRML). Figure 14 shows the comparison results of boxplot of WM, GM, CSF, and overall weighted average Dice ratio. It can be observed that using the hierarchical multi-level graph set strategy achieves the best registration accuracy compared with HUGS and HMRML. Although using full strategy the performance of registering CSF is relatively less accurate than HUGS method, our HMRML method still achieves significant overall better registration results compared to HUGS (see Fig. 12). Moreover, as illustrated in Fig. 15, using full strategy can dramatically reduce the computational cost compared to the HUGS method, where the total computation time is about 25 times faster. This significant efficiency improvement can be attributed to the following three reasons: (1) relatively simple registration within clusters and (2) simplified registration across clusters, since only a few exemplar images are involved. (3) the coarse level (low resolution) groupwise registration can provide a good initialization for the fine level (original resolution) registration task, which avoids registering images at original resolution from the scratch. It is worth noting that, in the current study, we only employed the original image intensity feature to calculate the similarity matrix *S* for AP clustering. Future works could include more image features, such as shape features, to improve the similarity measurement, yielding a better image clustering result to further improve the final registration.

## Conclusion

We have proposed to perform groupwise registration effectively and efficiently by registering the images from the bottom to the top of a hierarchical graph set, which captures image distribution within and between image clusters. Computational efficiency is improved with a multi-resolution strategy. Evaluations with two publicly available datasets show that our method yields promising results in terms of accuracy and speed.
